# Update: Cytokine Dysregulation in Chronic Nonbacterial Osteomyelitis (CNO)

**DOI:** 10.1155/2012/310206

**Published:** 2012-05-21

**Authors:** Sigrun R. Hofmann, Angela Roesen-Wolff, Gabriele Hahn, Christian M. Hedrich

**Affiliations:** ^1^Department of Pediatrics, University Hospital Carl Gustav Carus, Fetscherstra*β*e 74, 01307 Dresden, Germany; ^2^Department of Pediatric Radiology, University Hospital Carl Gustav Carus, 01307 Dresden, Germany; ^3^Division of Rheumatology, Department of Medicine, Beth Israel Deaconess Medical Center, Harvard Medical School, Boston, MA 02215, USA

## Abstract

Chronic nonbacterial osteomyelitis (CNO) with its most severe form chronic recurrent multifocal osteomyelitis (CRMO) is a non-bacterial osteitis of yet unknown origin. Secondary to the absence of both high-titer autoantibodies and autoreactive T lymphocytes, and the association with other autoimmune diseases, it was recently reclassified as an autoinflammatory disorder of the musculoskeletal system. Since its etiology is largely unknown, the diagnosis is based on clinical criteria, and treatment is empiric and not always successful. In this paper, we summarize recent advances in the understanding of possible etiopathogenetic mechanisms in CNO.

## 1. Introduction

Chronic nonbacterial osteomyelitis (CNO) (OMIM number 259680) is an autoinflammatory, noninfectious disorder that affects the skeletal system and has first been described in 1972 [1]. An association with further autoimmune and autoinflammatory disorders such as pustolusis palmoplantaris [2], chronic inflammatory bowel disease (IBD), psoriasis, c-ANCA positive vasculitis, Takayasu's arteriitis, and deficiency of IL-1 receptor antagonist (DIRA) has been discussed [3–5]. CNO covers a wide clinical spectrum from non- or oligo-symptomatic monofocal bony lesions to its most severe form chronic recurring multifocal osteomyelitis (CRMO). Generally, any skeletal site can be affected. However, metaphyses of long bones are predominantly involved [6] with a predilection for the lower extremities (distal femur, proximal tibia, distal tibia, and distal fibula [7]), followed by the clavicle and spine. A symmetric involvement is common. Systemic symptoms occur in a subset of patients and include low-grade fevers and malaise. Even though Scully et al. (2008) [7] reported a predominance in females (up to 85%), larger studies did not confirm this.

However, little is known about the pathophysiology of CNO. Most research articles focus on clinical aspects of CNO, and only few reports discuss putative pathomechanisms underlying this autoinflammatory disorder.

## 2. Classification, Clinical Picture, Diagnosis, and Treatment

Autoinflammatory diseases are rare disorders, characterized by recurrent episodes of fever and inflammation, and mostly affect joints, serosal surfaces, eyes and skin in the absence of high-titer autoantibodies, autoreactive T lymphocytes [11], and underlying infection [7]. In 2006, McGonagle and McDermott [12] suggested a clinical continuum with autoimmune disorders on the one, and autoinflammatory diseases on the other end of the spectrum. Disorders located towards the middle of the continuum exhibit features of both autoinflammatory as well as autoimmune diseases. According to this definition, CNO has been classified as an autoinflammatory disorder. In CNO the diagnosis relies on clinical symptoms, systemic inflammation, and elevated acute phase proteins during attacks. In very few cases, underlying mutations in the relevant genes can be detected (Majeed syndrome, see below). CNO needs to be differentiated from infectious osteomyelitis, including mycobacterial infections, and malignant neoplasms.

**Figure 1:**
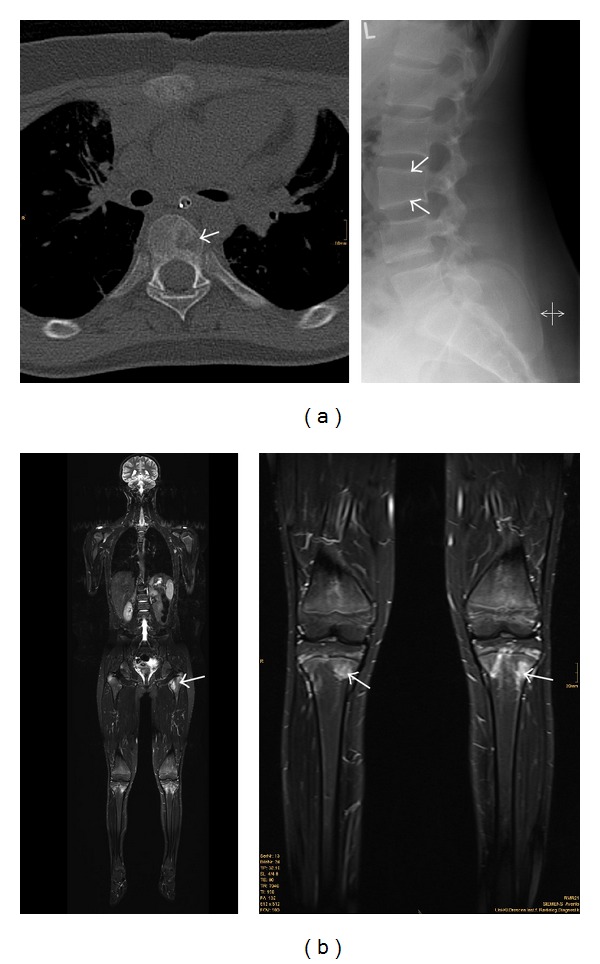
(a) left panel: computerized tomography (CT) of the spine, showing an osteolytic lesions (white arrow) in chronic recurrent multifocal osteomyelitis in a thoracic vertebra. Right panel: radiograph of the spine, showing flattening of the third lumbar vertebra (white arrows) in chronic recurrent multifocal osteomyelitis. (b) magnetic resonance imaging (MRI) in a patient with chronic recurrent multifocal osteomyelitis; left panel: whole-body MRI, showing multiple foci of osteomyelitis, some of which are distributed symmetrically; right panel: magnified image (from the left panel) of the knees, showing inflammatory epiphyseal lesions in both tibiae.

Laboratory and histopathological findings are nonspecific, with mildly elevated acute phase markers, and plasmacellular, granulocytic, and monocytic infiltrations respectively [13]. However in a substantial number of patients, systemic inflammatory signs (such as low-grade fever and malaise and elevated acute phase proteins) cannot be found, even during attacks. In these cases, the diagnosis of CNO can be made, combining clinical findings (localized bone pain, swelling, and warmth in the affected areas) and laboratory tests (including testing for mycobacteria) with radiographic techniques. The presence of osteolytic lesions with surrounding sclerosis in X-ray imaging, supplemented by computerized tomography (CT) and/or magnetic resonance imaging (MRI), is usually helpful (Figure 1). Silent asymptomatic lesions frequently appear on nuclear scans.

Even though CNO has been treated with azithromycin [14] in the past, it does generally not respond to antibiotics. Treatment involves anti-inflammatory agents, including NSAIDs [13, 15, 16] and corticosteroids. In treatment restistant cases, bisphosphonates [17] and biologicals, usually infliximab or etanercept (blocking tumor necrosis factor-*α*) [18] have been applied successfully, suggesting an involvement of TNF-*α* in the pathophysiology of CNO.

Individual clinical courses of CNO are generally unpredictable. Most cases are self-limiting and eventually resolve without major sequelae. However, a subset of patients exhibit severe and prolonged disease courses and are at risk of developing bone deformities that can be caused by premature closure of the epiphyses, and kyphoscoliosis as a result of vertebral involvement [4, 6, 19]. Disease onset at a young age and high numbers [20] of inflammatory bone lesions at disease onset seem to be predictive of poor outcomes [4, 19].

## 3. Putative Pathomechanisms

Imbalances in cytokine expression can cause a disruption of immune homeostasis, resulting in increased or reduced inflammatory responses that in turn may cause susceptibility to infections or autoimmune disorders. Cytokine dysregulation can be caused on various levels, including (1) impaired transcription that can be caused by genomic variation and (2) epigenetic modifications. Genomic variations, including single-nucleotide polymorphisms (SNPs), can influence gene expression by variable transcription factor binding or variation in genome organization [21]. Epigenetic modifications alter chromatin accessibility for transcription factors and/or RNA polymerases [21, 22]. Posttranslational histone modifications influence the transcriptional activity of genes and serve as markers of epigenetic changes. Histone hypermethylation as repressing histone modification mediates dense packing of nucleosomes in transcriptionally silent, so-called “heterochromatic” regions. Activating histone modifications, such as histone acetylation and phosphorylation, mediate “open” or “euchromatic” chromatine conformation that is accessible for transcriptional regulatory factors and RNA polymerases [8].

### 3.1. Genetic Susceptibility Studies

Genetic studies suggested a predisposing locus for CNO on chromosome 18q21.3-18q22 [23]. However, convincing evidence for the involvement of this locus in the pathophysiology of CNO is still lacking. Several observations suggest the contribution of genetic factors to the etiology of CNO.

Ferguson et al. [24, 25] described homozygous mutations in the *LPIN2* gene, causing a syndromic autosomal recessive form of CNO known as Majeed syndrome. The Majeed syndrome's classical triad includes early-onset CRMO, congenital dyserythropoietic anemia and a neutrophilic dermatosis. It is an extremely rare disease and has only been identified in three unrelated Arabic families [14, 26]. Bone lesions in Majeed syndrome occur within the first two years of life, and the course is more aggressive then in CNO, with more relapses. *LPIN2* was mapped to chromosome 18p and found to be expressed in almost all tissues [25]. Little is known about the function of *LPIN2*. It shares a lipin domain with the human ortholog of murine *lpin1*, which plays a role in fat metabolism. Because of the dyserythropoesis in Majeed syndrome, it has been proposed that *LPIN2* could also play a role in mitosis, and that an impaired function may lead to abnormal mitosis in rapidly dividing cells, including the red blood cell lineage in the bone marrow [25]. It has further been hypothesised that mutations in *LPIN2* may play an etiologic role in psoriasis, since it localizes to a genomic region identified as a psoriasis susceptibility locus. Furthermore, all carriers of disease causing homozygous *LPIN2* mutations exhibit inflammatory dermatoses [25].

**Figure 2:**
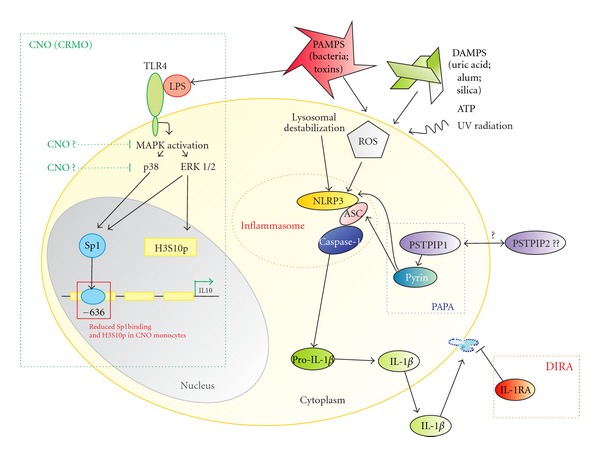
Possible pathophysiological mechanisms in autoinflammatory diseases with emphasis on CNO. Modified from [8, 9]. Endogenous and exogenous danger signals (danger-associated molecular patterns: DAMPs and pathogen-associated molecular patterns: PAMPs) activate the NLRP3 inflammasome that is involved in various autoinflammatory disorders. The exact pathways that result in NLRP3 activation are yet unknown. However, reactive oxygen species (ROS) and lysosomal destabilization seem to be involved. The selected monogenic autoinflammatory syndromes result in an activation of the caspase-1 complex. In *PAPA*, mutations in *PSTPIP1 *lead to prolonged binding of PSTPIP1 to pyrin via an SH3 domain [10] and impairment of pyrin function, resulting in constitutive activation of NLRP3, production of IL-1*β*, and inflammation. IL-1 receptor antagonist (IL-1RA) is a naturally occurring IL-1*β* antagonist, and mutations in the *IL1RA* gen lead to *DIRA*. Based on the finding that Sp1 binding to the IL10 promoter is reduced, and H3S10 phosphorylation is impaired in the same region in monocytes from *CNO* patients, we concluded that processes upstream of Sp1 activation and H3S10 phosphorylation may be involved. A reduced activity of MAP kinases upstream of Sp1 signalling may be responsible, resulting in reduced Sp1 activation and reduced H3S10 phosphorylation of the IL10 proximal promoter. ASC: apoptosis-associated speck-like protein containing a caspase recruitment domain; Casp1: enzymatically active caspase-1; IL-18: interleukin-18; IL-1*β*: interleukin-1*β*; NLRP3: NOD-like receptor family, pyrin domain containing 3; pro-Casp1: procaspase-1; ROS: reactive oxygen species; CNO: chronic nonbacterial osteomyelitis; DIRA: deficiency in IL-1 receptor antagonist; PAPA: pyogenic arthritis, pyoderma gangrenosum and acne; PSTPIP1: proline-serine-threonine phosphatase-interacting protein 1.

Mutations in the *pstpip2* gene have been described in the murine form of CNO [27]. To date, there are two murine CNO models, both carrying mutations in the *pstpip2* gene: (1) cmo mice [26–29] and (2) Lupo mice [30]. Cmo (chronic multifocal osteomyelitis) mice have been reported to develop a phenotype that is similar to severe cases of human CNO with multiple sites of inflammation. However, most CNO patients present with less severe mono- or oligo-focal disease which does not completely resemble the disease in cmo mice that develop multifocal (systemic) inflammatory symptoms. The disease is inherited in an autosomal recessive manner, and the cmo locus has been mapped to the murine chromosome 18 [27]. Within the refined region was the gene *pstpip2*, which shares significant sequence homology to human *PSTPIP1*. Expression patterns of murine Pstpip2, also called MAYP (macrophage actin-associated tyrosine-phosphorylated protein), are discussed controversially. While Pstpip2 mRNA is ubiquitously expressed [31], Western blot analysis revealed narrow protein expression patterns, with the highest Pstpip2 expression in myeloid cells [32]. Pstpip2 plays a role in cytoskeletal reorganization, and localizes to the cytosol as an F-actin associated phosphoprotein that interacts with PEST-type protein tyrosine phosphatases (PTP PEST) [31, 32]. The association with PEST family PTPs inhibits tyrosine phosphorylation of wildtype PSTPIP1 [33]. Mutations in the *PSTPIP1* gene have been documented to cause the autoinflammatory PAPA syndrome (pyogenic arthritis, pyoderma gangrenosum, and acne). The PSTPIP1 protein binds to pyrin, a regulator that inhibits the NLRP3 inflammasome. Interestingly, Veillette et al. [33] and Wise et al. [34] demonstrated that the *PSTPIP1* mutations found in the autoinflammatory PAPA syndrome are in the putative coil-coil domain of PSTPIP1, abolishing the capacity of PSTPIP1 to bind PEST family PTPs. This alteration results in enhanced PSTPIP1 tyrosine phosphorylation and is accompanied by augmented binding of PSTPIP1 to pyrin via an SH3 domain [10], resulting in constitutive activation of NLRP3, production of IL-1*β*, and inflammation [33] (Figure 2). Some similarities in the phenotypes of cmo mice and (human) PAPA patients may suggest an involvement of the same or similar immunologic pathways. However, the absence of the SH3 domain in Pstpip2 suggests that it does not bind pyrin directly [27]. However, “classical” CNO patients do not develop the characteristic pustulotic or pyogenic dermatitis that is seen in PAPA patients.

Even though cmo mice serve as an interesting model for bone inflammation, none of the reported CNO patients [35] carry mutation in the *PSTPIP1* and *PSTPIP2* genes, except for several known polymorphisms.

The recent report of a condition, presenting with neonatal onset sterile multifocal osteomyelitis, periostosis, and pustulosis, that is caused by a deficiency of IL-1 receptor antagonist (*DIRA*), is of special interest [5]. Because of similar clinical, radiological and histological features of CNO and DIRA, both disorders might share common or similar autoinflammatory processes. However, in a recent study [36] a heterozygous missense mutation c.281G>T (p.Cys94Phe) could only be found in one patient with CNO. In the other patients, only frequent polymorphisms were found.

Another hint towards the understanding of genetic contributors to the pathophysiology of CNO may be the clinical similarities of CNO patients and patients with hypophosphatasia [37, 38]. Hypophosphatasia is a rare defect of bone metabolism that is caused by a number of mutations in the gene encoding for the tissue-nonspecific alkaline phosphatase (*TNSALP*) on chromosome 1p36.1. The mode of inheritance is mostly autosomal-recessive [37, 39, 40]. *TNSALP* mutations result in reduced enzyme activity and elevated concentrations of TNSALP substrates, including inorganic pyrophosphate, pyridoxal-50-phosphate and phosphoethanolamine [41–43]. It has been documented that pyrophosphate crystals stimulate the NLRP3 inflammasome resulting in Interleukin-1 production [44]. An inflammatory process secondary to the metabolic defect in hypophosphatasia involving the NLRP3 inflammasome may therefore be involved in the pathogenesis of CNO.

Since associations between CNO and Crohn's disease, a multifactorial autoinflammatory disease, have been reported [26], Morbach and colleagues [45] analyzed CNO patients for NOD2/CARD15 mutations. However, CNO without intestinal inflammation was not associated with common *CARD15 *gene variants [45].

In addition, susceptibility regions have been identified on chromosome 18 in two other diseases sharing similarities with CNO: familial expansile osteolysis and familial Paget's disease of bone [26, 46, 47]. The phenotypes of both diseases have been linked to mutations in the *TNFRSF11A* gene at 18q22.1, which encodes the receptor activator of nuclear factor kappa B (RANK) [46, 47].

Recently, we reported an association of CNO with *IL10* promoter polymorphisms [3, 8]. IL-10 is an immune-regulatory cytokine that controls inflammation by limiting inflammatory cytokine expression and antigen presentation. Dysregulation in IL-10 expression, sometimes caused by single-nucleotide polymorphisms (SNPs) in the IL10 promoter have been associated to autoimmune and infectious diseases, such as SLE [48], asthma, chronic viral diseases, rheumatoid arthritis as well as transplantation complications and cancer [49–51].

IL10 promoter polymorphisms as an example for genomic variation, appear to play an important role in the interindividual variation of IL-10 expression [8, 52]. The IL10 promoter polymorphisms −1082G>A (rs1800869), −819C>T (rs1800871), and −592C>A (rs1800872) are in tight linkage disequilibrium and form three well-defined or “classical” haplotypes: ATA, ACC, and GCC [3]. ATA is associated with low, ACC with intermediate, and GCC with high levels of IL-10 expression [48, 53, 54]. However, for the interindividual variability of IL-10 expression, additional host factors seem to play an important role [52].

As aforementioned, an association of IL10 promoter polymorphisms with CNO has been described [8], demonstrating an increased frequency of high IL-10 expressing −1082G/G alleles, and resulting GCC haplotypes. Interestingly, monocytes from CNO patients failed to produce IL-10 in response to stimulation with LPS. This could be explained by (1) *IL10* promoter polymorphisms as part of larger haplotypes, extending several hundred kilobases over the entire cytokine cluster, develop cumulative effects in a genomic setting [8]. This hypothesis is supported by a recent study [55] that failed to find a link between IL10 promoter SNPs and inflammatory bowel disease (IBD), but showed an association between a SNP 3′ to the *IL10* gene and ulcerative colitis. (2) since it is known that IL-10 mediates the proliferation of various lymphocytic tissues, including B cells, and enhances antibody production [56]. Lymphocytes, but also plasma cells can be predominantly found in biopsies, taken from CNO lesions. Having the anti-inflammatory aspects of IL-10 expression in mind, an association with GCC haplotypes could partly explain the lymphocyte expansion in affected tissues [6]. (3) additional, namely, epigenetic mechanisms, could be involved in the dysregulation of cytokine expression in CNO (see below).

### 3.2. Epigenetic Mechanisms

The expression of pro- and anti-inflammatory cytokines, including IL-10, is considered to be under epigenetic control. Zhang et al. [57] reported histone-phosphorylation within the *IL10* proximal promoter to be associated with transcription. Histone phosphorylation is particularly increased in regulatory regions that bind the transcription factor specificity protein 1 (Sp1) and 3 (Sp3) and mediate transcriptional activation in response to stimulation with LPS [58–61]. We recently demonstrated that monocytes from CNO patients fail to produce IL-10. Since the proximal promoter region of *IL10* displays multiple GC rich sequences, which bind SP-family transcription factors, the binding of the transcriptional regulatory factor Sp1 (previously reported to play a role in the regulation of IL-10 in monocytes and macrophages [55]) to the *IL10* promoter has been investigated. A markedly reduced Sp1 recruitment to the *IL10_*636 Sp1 element has been detected in monocytes from CNO patients in response to LPS stimulation [8]. This was accompanied with reduced histone H3 serine 10 phosphorylation (H3S10p), an activating epigenetic mark. These findings indicate (1) reduced Sp1 recruitment and (2) attenuated H3S10 phosphorylation around the *IL10_*636 element within the *IL10* promoter [8].

Given that (1) LPS stimulation induces MAP kinase (MAPK) activation in monocytes and macrophages, including the p38 [61], the ERKs 1 and 2, and the JNK MAPK pathway [58], and (2) ERKs 1 and 2 are implicated in H3S10 phosphorylation [62], these reports could indicate a new pathophysiological mechanism in CNO with impaired MAPK signaling, resulting in reduced H3S10 phosphorylation, and Sp1 recruitment to the *IL10* promoter and subsequently attenuated gene expression and immune homeostasis [8].

### 3.3. Other Possible Etiopathogenetic Mechanisms of CNO

TNF-*α* and IL-1*β* are implicated in the pathophysiology of various autoimmune and autoinflammatory disorders, including CNO, where it has been documented to be increased in inflammatory lesions [35]. Furthermore, clinical response to anti-TNF*α* or IL-1 receptor antagonist (IL-1ra) anakinra treatment has been documented in CNO (summarized in [63]). However, it remains unclear whether increased TNF-*α* and IL-1*β* expression is the pathophysiological cause of CNO or a downstream event in the disease. The aforementioned reduced expression of IL-10 in monocytes from CNO patients may well contribute to a cytokine imbalance towards proinflammatory signals, including TNF-*α*.

## 4. Conclusions

Only few CNO cases have been associated with mutations in single genes. However, they do not explain the pathophysiology of CNO in the vast majority of cases. Thus, neither the etiology nor the pathogenesis of CNO is completely understood. Mutations in the *TNFα* and *IL1RA* genes in humans and the *Pstpip2* gene in mice appear to be promising in the search for pathomechanisms in CNO. The most recently described pathophysiological mechanism in CNO might involve impaired MAPK signaling, reduced H3S10 phosphorylation, and attenuated Sp1 recruitment to the *IL10* promoter that results in an impaired gene expression.

## References

[1] Giedion A, Holthusen W, Masel LF, Vischer D (1972). Subacute and chronic “symmetrical” osteomyelitis. *Annales de Radiologie*.

[2] Bjorksten B, Gustavson KH, Eriksson B, Lindholm A, Nordström S (1978). Chronic recurrent multifocal osteomyelitis and pustulosis palmoplantaris. *Journal of Pediatrics*.

[3] Hamel J, Paul D, Gahr M, Hedrich CM (2011). Pilot study: possible association of *IL10* promoter polymorphisms with CRMO. *Rheumatology International*.

[4] Iyer RS, Thapa MM, Chew FS (2011). Chronic recurrent multifocal osteomyelitis: review. *American Journal of Roentgenology*.

[5] Aksentijevich I, Masters SL, Ferguson PJ (2009). An autoinflammatory disease with deficiency of the interleukin-1-receptor antagonist. *The New England Journal of Medicine*.

[6] Jurik AG (2004). Chronic recurrent multifocal osteomyelitis. *Seminars in Musculoskeletal Radiology*.

[7] Scully C, Hodgson T, Lachmann H (2008). Auto-inflammatory syndromes and oral health. *Oral Diseases*.

[8] Hofmann SR, Schwarz T, Moller JC, Morbach H, Schnabel A (2011). Chronic non-bacterial osteomyelitis is associated with impaired Sp1 signaling, reduced *IL10* promoter phosphorylation, and reduced myeloid *IL10* expression. *Clinical Immunology*.

[9] Hofmann SR, Heymann MC, Hermsdorf A, Roesen-Wolff A (2011). Recent advances in autoinflammatory diseases and animal models. *Journal of Genetic Syndromes & Gene Therapy*.

[10] Shoham NG, Centola M, Mansfield E (2003). Pyrin binds the PSTPIP1/CD2BP1 protein, defining familial Mediterranean fever and PAPA syndrome as disorders in the same pathway. *Proceedings of the National Academy of Sciences of the United States of America*.

[11] Kastner DL, Aksentijevich I, Goldbach-Mansky R (2010). Autoinflammatory disease reloaded: a clinical perspective. *Cell*.

[12] McGonagle D, McDermott MF (2006). A proposed classification of the immunological diseases. *PLoS Medicine*.

[13] Girschick HJ, Zimmer C, Klaus G, Darge K, Dick A, Morbach H (2007). Chronic recurrent multifocal osteomyelitis: what is it and how should it be treated?. *Nature Clinical Practice Rheumatology*.

[14] Twilt M, Laxer RM (2011). Clinical care of children with sterile bone inflammation. *Current Opinion in Rheumatology*.

[15] Weihe S, Eufinger H, Terhaar O, Konig M, Machtens E (2000). Mandibular involvement in chronic recurrent multifocal osteomyelitis (CRMO) in adulthood. *Mund Kiefer Gesichtschir*.

[16] Abril JC, Ramirez A (2007). Successful treatment of chronic recurrent multifocal osteomyelitis with indomethacin: a preliminary report of five cases. *Journal of Pediatric Orthopaedics*.

[17] Compeyrot-Lacassagne S, Rosenberg AM, Babyn P, Laxer RM (2007). Pamidronate treatment of chronic noninfectious inflammatory lesions of the mandible in children. *Journal of Rheumatology*.

[18] Deutschmann A, Mache CJ, Bodo K, Zebedin D, Ring E (2005). Successful treatment of chronic recurrent multifocal osteomyelitis with tumor necrosis factor-*α* blockage. *Pediatrics*.

[19] Iyer RS, Thapa MM, Chew FS (2011). Imaging of chronic recurrent multifocal osteomyelitis: self-assessment module. *American Journal of Roentgenology*.

[20] Catalano-Pons C, Comte A, Wipff J (2008). Clinical outcome in children with chronic recurrent multifocal osteomyelitis. *Rheumatology*.

[21] Hedrich CM, Bream JH (2010). Cell type-specific regulation of IL-10 expression in inflammation and disease. *Immunologic Research*.

[22] Brenner C, Fuks F (2007). A methylation rendezvous: reader meets writers. *Developmental Cell*.

[23] Golla A, Jansson A, Ramser J (2002). Chronic recurrent multifocal osteomyelitis (CRMO): evidence for a susceptibility gene located on chromosome 18q21.3–18q22. *European Journal of Human Genetics*.

[24] El-Shanti HI, Ferguson PJ (2007). Chronic recurrent multifocal osteomyelitis: a concise review and genetic update. *Clinical Orthopaedics and Related Research*.

[25] Ferguson PJ, Chen S, Tayeh MK (2005). Homozygous mutations in LPIN2 are responsible for the syndrome of chronic recurrent multifocal osteomyelitis and congenital dyserythropoietic anaemia (Majeed syndrome). *Journal of Medical Genetics*.

[26] Wipff J, Adamsbaum C, Kahan A, Job-Deslandre C (2011). Chronic recurrent multifocal osteomyelitis. *Joint Bone Spine*.

[27] Ferguson PJ, Bing X, Vasef MA (2006). A missense mutation in pstpip2 is associated with the murine autoinflammatory disorder chronic multifocal osteomyelitis. *Bone*.

[28] Byrd L, Grossmann M, Potter M, Shen-Ong GL (1991). Chronic multifocal osteomyelitis, a new recessive mutation on chromosome 18 of the mouse. *Genomics*.

[29] Hentunen TA, Choi SJ, Boyce BF (2000). A murine model of inflammatory bone disease. *Bone*.

[30] Grosse J, Chitu V, Marquardt A (2006). Mutation of mouse Mayp/Pstpip2 causes a macrophage autoinflammatory disease. *Blood*.

[31] Wu Y, Dowbenko D, Lasky LA (1998). PSTPIP 2, a second tyrosine phosphorylated, cytoskeletal-associated protein that binds a PEST-type protein-tyrosine phosphatase. *The Journal of Biological Chemistry*.

[32] Yeung YG, Soldera S, Stanley ER (1998). A novel macrophage actin-associated protein (MAYP) is tyrosine- phosphorylated following colony stimulating factor-1 stimulation. *The Journal of Biological Chemistry*.

[33] Veillette A, Rhee I, Souza CM, Davidson D (2009). PEST family phosphatases in immunity, autoimmunity, and autoinflammatory disorders. *Immunological Reviews*.

[34] Wise CA, Gillum JD, Seidman CE (2002). Mutations in CD2BP1 disrupt binding to PTP PEST and are responsible for PAPA syndrome, an autoinflammatory disorder. *Human Molecular Genetics*.

[35] Jansson A, Renner ED, Ramser J (2007). Classification of non-bacterial osteitis: retrospective study of clinical, immunological and genetic aspects in 89 patients. *Rheumatology*.

[36] Beck C, Girschick HJ, Morbach H, Schwarz T, Yimam T (2011). Mutation screening of the IL-1 receptor antagonist gene in chronic non-bacterial osteomyelitis of childhood and adolescence. *Clinical and Experimental Rheumatology*.

[37] Whyte MP, Wenkert D, McAlister WH (2009). Chronic recurrent multifocal osteomyelitis mimicked in childhood hypophosphatasia. *Journal of Bone and Mineral Research*.

[38] Beck C, Morbach H, Richl P, Stenzel M, Girschick HJ (2009). How can calcium pyrophosphate crystals induce inflammation in hypophosphatasia or chronic inflammatory joint diseases?. *Rheumatology International*.

[39] Beck C, Morbach H, Stenzel M (2009). Hypophosphatasia. *Klinische Padiatrie*.

[40] Girschick HJ, Mornet E, Beer M, Warmuth-Metz M, Schneider P (2007). Chronic multifocal non-bacterial osteomyelitis in hypophosphatasia mimicking malignancy. *BMC Pediatrics*.

[41] Mornet E (2007). Hypophosphatasia. *Orphanet Journal of Rare Diseases*.

[42] Brun-Heath I, Lia-Baldini AS, Maillard S (2007). Delayed transport of tissue-nonspecific alkaline phosphatase with missense mutations causing hypophosphatasia. *European Journal of Medical Genetics*.

[43] Beck C, Morbach H, Wirth C, Beer M, Girschick HJ (2011). Whole-body MRI in the childhood form of hypophosphatasia. *Rheumatology International*.

[44] Pazar B, Ea HK, Narayan S (2011). Basic calcium phosphate crystals induce monocyte/macrophage IL-1*β* secretion through the NLRP3 inflammasome in vitro. *Journal of Immunology*.

[45] Morbach H, Dick A, Beck C (2010). Association of chronic non-bacterial osteomyelitis with Crohn’s disease but not with CARD15 gene variants. *Rheumatology International*.

[46] Hughes AE, Ralston SH, Marken J (2000). Mutations in TNFRSF11A, affecting the signal peptide of RANK, cause familial expansile osteolysis. *Nature Genetics*.

[47] Sparks AB, Peterson SN, Bell C (2001). Mutation screening of the TNFRSF11A gene encoding receptor activator of NFkB (RANK) in familial and sporadic Paget’s disease of bone and osteosarcoma. *Calcified Tissue International*.

[48] Gibson AW, Edberg JC, Wu J, Westendorp RG, Huizinga TW, Kimberly RP (2001). Novel single nucleotide polymorphisms in the distal IL-10 promoter affect IL-10 production and enhance the risk of systemic lupus erythematosus. *Journal of Immunology*.

[49] Eskdale J, Wordsworth P, Bowman S, Field M, Gallagher G (1997). Association between polymorphisms at the human IL-10 locus and systemic lupus erythematosus. *Tissue Antigens*.

[50] Lazarus R, Vercelli D, Palmer LJ (2002). Single nucleotide polymorphisms in innate immunity genes: abundant variation and potential role in complex human disease. *Immunological Reviews*.

[51] Vicari AP, Trinchieri G (2004). Interleukin-10 in viral diseases and cancer: exiting the labyrinth?. *Immunological Reviews*.

[52] Reuss E, Fimmers R, Kruger A, Becker C, Rittner C, Höhler T (2002). Differential regulation of interleukin-10 production by genetic and environmental factors—a twin study. *Genes and Immunity*.

[53] Mormann M, Rieth H, Hua TD (2004). Mosaics of gene variations in the interleukin-10 gene promoter affect interleukin-10 production depending on the stimulation used. *Genes and Immunity*.

[54] Salhi A, Rodrigues V, Santoro F (2008). Immunological and genetic evidence for a crucial role of *IL10* in cutaneous lesions in humans infected with Leishmania braziliensis. *Journal of Immunology*.

[55] Franke A, Balschun T, Karlsen TH (2008). Sequence variants in *IL10*, ARPC2 and multiple other loci contribute to ulcerative colitis susceptibility. *Nature Genetics*.

[56] Hedrich CM, Ramakrishnan A, Dabitao D, Wang F, Ranatunga D, Bream JH (2010). Dynamic DNA methylation patterns across the mouse and human *IL10* genes during CD4^+^ T cell activation; influence of IL-27. *Molecular Immunology*.

[57] Zhang X, Edwards JP, Mosser DM (2006). Dynamic and transient remodeling of the macrophage IL-10 promoter during transcription. *Journal of Immunology*.

[58] Brightbill HD, Plevy SE, Modlin RL, Smale ST (2000). A prominent role for Sp1 during lipopolysaccharide-mediated induction of the IL-10 promoter in macrophages. *Journal of Immunology*.

[59] Ma W, Lim W, Gee K (2001). The p38 mitogen-activated kinase pathway regulates the human interleukin-10 promoter via the activation of Sp1 transcription factor in lipopolysaccharide-stimulated human macrophages. *The Journal of Biological Chemistry*.

[60] Steinke JW, Barekzi E, Hagman J, Borish L (2004). Functional analysis of -571 IL-10 promoter polymorphism reveals a repressor element controlled by Sp1. *Journal of Immunology*.

[61] Chanteux H, Guisset AC, Pilette C, Sibille Y (2007). LPS induces IL-10 production by human alveolar macrophages via MAPKinases- and Sp1-dependent mechanisms. *Respiratory Research*.

[62] Lucas M, Zhang X, Prasanna V, Mosser DM (2005). ERK activation following macrophage Fc*γ*R ligation leads to chromatin modifications at the IL-10 locus. *Journal of Immunology*.

[63] Eleftheriou D, Gerschman T, Sebire N, Woo P, Pilkington CA, Brogan PA (2010). Biologic therapy in refractory chronic non-bacterial osteomyelitis of childhood. *Rheumatology*.

